# Cross-sectional association among dietary habits, periodontitis, and uncontrolled diabetes in Hispanics: the LLIPDS study

**DOI:** 10.3389/froh.2025.1468995

**Published:** 2025-01-31

**Authors:** Hunter Smith, David Travis Thomas, Gabriela Nicole Vázquez-Morales, Lakin Puckett, María Del Mar Rodriguez, Arnold Stromberg, Luciana Macchion Shaddox, Mauro Pedrine Santamaria, Kevin Pearce, Oelisoa Mireille Andriankaja

**Affiliations:** ^1^ College of Arts & Sciences, University of Kentucky, Lexington, KY, United States; ^2^ Department of Athletic Training and Clinical Nutrition, College of Health Sciences, University of Kentucky, Lexington, KY, United States; ^3^ Natural Sciences Department, University of Puerto Rico, Cayey, Puerto Rico; ^4^ Department of Dietetics and Human Nutrition, College of Agriculture, Food, & Environment, University of Kentucky, Lexington, KY, United States; ^5^ School of Dental Medicine, Center for Clinical Research and Health Promotion, Medical Sciences Campus, University of Puerto Rico, San Juan, Puerto Rico; ^6^ Dr. Bing Zhang Department of Statistics, College of Arts & Sciences, University of Kentucky, Lexington, KY, United States; ^7^ College of Dentistry, Center for Oral Health Research, University of Kentucky, Lexington, KY, United States; ^8^ Department of Family and Community Medicine, College of Medicine, University of Kentucky, Lexington, KY, United States

**Keywords:** dietary habits, periodontal disease, glucose control, type 2 diabetes, comorbidity, risk factor

## Abstract

**Objectives:**

Type 2 Diabetes (T2D) is recognized as a risk factor for periodontal disease (PD), with evidence supporting a bidirectional relationship. Food choices are thought to influence both conditions, but research on their impact specifically on PD remains limited. This study aimed to explore whether food choices were linked to higher prevalence of adverse periodontal parameters and poorly controlled glucose levels among Hispanic adults with T2D.

**Methods:**

A cross-sectional study was conducted with 260 Puerto Rican adults aged 40–65 years, all diagnosed with T2D. Dietary habits were assessed by weekly frequencies of food choices deemed healthy or unhealthy over the past year. Periodontal health was evaluated by the percentage of sites with probing pocket depth (PPD) ≥4 mm and bleeding on probing (BOP) at corresponding teeth. Glucose control was measured by glycated hemoglobin (HbA1c) levels, with uncontrolled glucose defined as HbA1c ≥ 7%. Linear regression models adjusted for demographic and clinical variables estimated associations with PD. Logistic regression assessed associations with glucose control.

**Results:**

The median Healthy Eating Score was 0.5 (Q1, Q3: −3.9, 4.5). A higher Healthy Eating Score was significantly associated with fewer sites exhibiting PPD ≥ 4 mm and BOP (adjusted β: −0.02; SE: 0.01; *p* = 0.035), and reduced odds of uncontrolled glucose (adjusted odds ratio: 0.94; 95% CI: 0.89–0.98; *p* = 0.007).

**Conclusions:**

Adherence to a healthier dietary pattern appears to correlate with lower periodontal inflammation and greater glucose control among Hispanics with T2D. Prospective studies are needed to confirm causality and long-term effects.

## 1 Introduction

### 1.1 Periodontal disease

Periodontal disease (PD) is a pathologic condition of the periodontal tissue responsible for supporting teeth in their place (1). It occurs from prolonged inflammation of the gingiva and bones surrounding them. In serious cases, this condition can also affect the integrity of the bones of the jaw. Between the years of 2009–2014, it was estimated that 42.2% of US adults between the ages of 30 and 79 had chronic PD (2). While the prevalence among Americans is high, there is a higher prevalence among Hispanic populations. A study conducted in Puerto Rico estimated a 44.5% prevalence rate of chronic PD in adults aged 70–97 years (3). Furthermore, PD is commonly seen in patients with other chronic conditions, such as Type 2 Diabetes (T2D). According to a study conducted by Singh et. al, some degree of periodontal destruction occurred in greater than 90% of T2D patients, aged 30–65 years (4).

### 1.2 Type II diabetes

Diabetes mellitus (DM) encompasses metabolic disorders characterized by elevated blood glucose levels, known as hyperglycemia. Type 2 diabetes (T2D), accounting for 90%–95% of DM cases, involves insufficient insulin secretion, impaired insulin action (insulin resistance), or both (5, 6). Insulin resistance reduces cells’ responsiveness to insulin, leading to chronic hyperglycemia and metabolic dysregulation in T2D (7). Long-term, T2D can lead to organ damage due to diabetes complications (8). PD is recognized to be the 6th complication of diabetes (9). Like periodontitis epidemiology, T2D prevalence is higher among Hispanic individuals living in the United States. Specifically, T2D prevalence in Hispanic adults is approximately 80% higher than non-Hispanic white adults in the US (10).

### 1.3 Type II diabetes and periodontal disease risk factors

Along with commonly appearing as comorbid conditions, T2D and PD also have similar risk factors (11). Increased risks for T2D and PD are thought to be influenced by factors such as household income, healthcare accessibility, and food insecurity (12–18). Other factors associated with these conditions are smoking, genetics, race/ethnicity, and physical activity (11, 19–24). While, unhealthy dietary choices are also established as a key factor in the etiology and treatment of T2D, there is currently a distinct gap in research regarding the effects of dietary choices on PD.

The National Health and Nutrition Examination Survey (NHANES) data from 2009 to 2014 has suggested that eating more fruit and vegetables with water or tea may result in less periodontitis (2). While this recommendation aligns well with the 2020–2025 Dietary Guidelines for Americans, a stronger understanding of the role that dietary choices may play in preventing or managing PD may offer a frontline treatment to improve oral health. Once PD is diagnosed, treatment can become complex and dietary recommendations are not clear. Periodontal treatment in individuals with T2D is rather challenging due to the patients being hyperglycemic and in a hyperinflammatory state. Consuming less refined carbohydrate may improve T2D symptoms, but the association between overall dietary choices on PD prevalence and treatment outcomes is not well established. Our primary objective was to examine the association between dietary choices and PD. Our secondary objective was to examine the association between dietary choices and glucose control among Hispanic adults diagnosed with T2D.

## 2 Materials and methods

### 2.1 Study population

**Figure 1:**
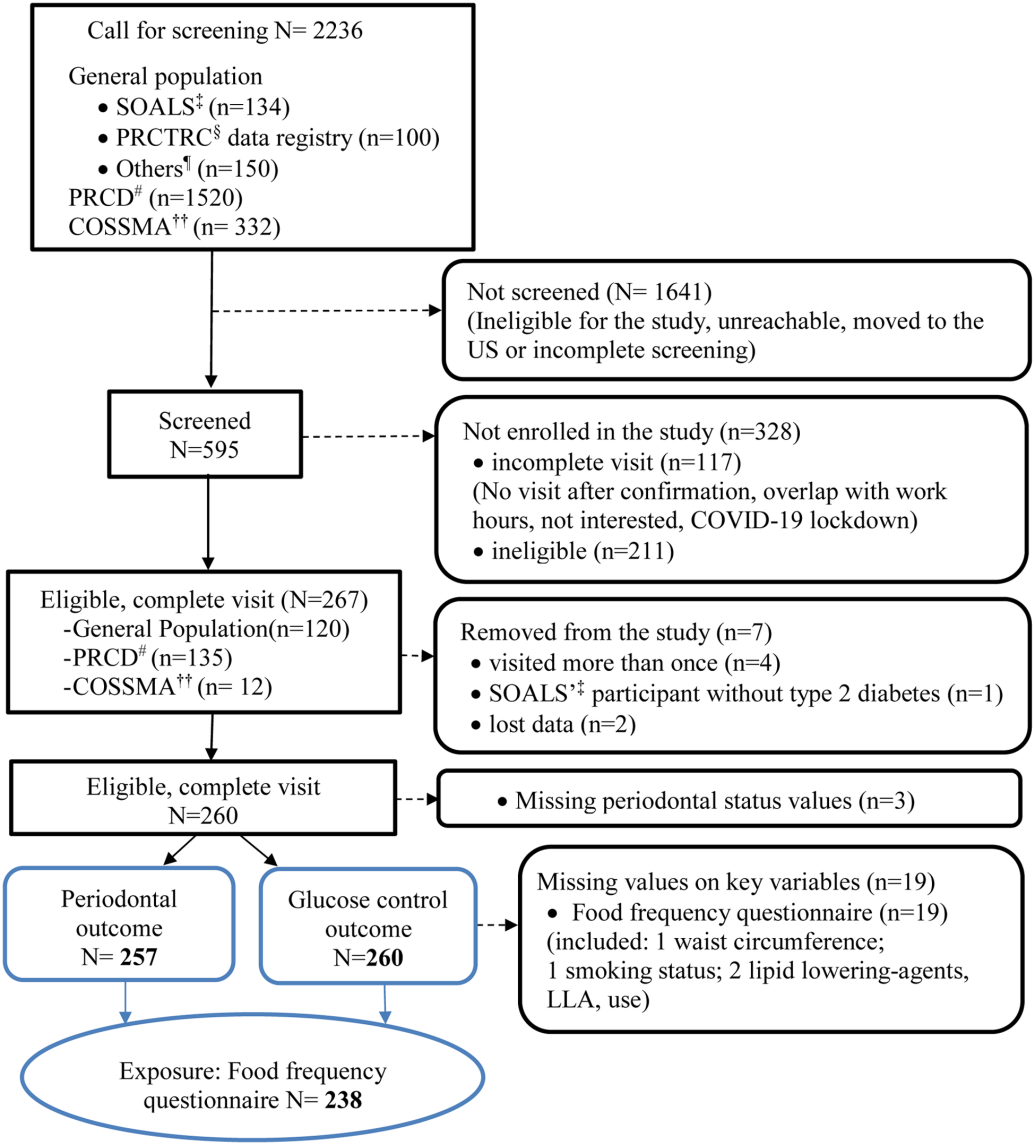
Diagram of LLIPDS's participants recruitment: April 26, 2017–March 9, 2020 (*N* = 260). **^†^LLIPDS**, lipid-lowering agents use in periodontitis and diabetes study; ^‡^**SOALS**, San Juan overweigh adults longitudinal study; ^§^**PRCTRC**, Puerto Rico clinical and translational research consortium; ^¶^**Others**, e.g., participants’ responses to the advertisements, words of mouths, etc; ^#^**PRCD**, Puerto Rico center for diabetes; **^††^COSSMA**, Inc.: A non-profit organization based in Humacao, Puerto Rico with six primary healthcare clinics serving the population throughout Puerto Rico Island.

Data were extracted and analyzed from the cross-sectional study titled “Lipid-Lowering agents use in Periodontitis and Diabetes Study” (6). Participants were recruited from three primary sources (see Figure 1): the Puerto Rico Center for Diabetes (PRCD) (50%), the general population (45%), including 7% from the San Juan Overweight Adults Longitudinal Study (SOALS), and COSSMA, a private decentralized healthcare organization in Puerto Rico (5%). The *Inclusion criteria* included: (a) age 40–65 years.; (b) had a minimum of four natural teeth to obtain an accurate periodontal measurement; (c) diagnosed with T2D by a physician, based on their medications, or through fasting blood glucose and glycosylated hemoglobin A1C (HbA1c) levels (See description below). The ***exclusion criteria*** included: (a) orthodontic appliances or significant oral pathology that could interfere with periodontal measurements; (b) orthodontic appliances or gross oral pathology that might impair periodontal measurements; (c) regularly use of antibiotics, immunosuppressants, steroids, or anti-inflammatory drugs (except aspirin at doses of ≤150 mg/day) before the screening; (d) took any medication known to affect periodontal health for two weeks or longer within one month preceding the clinical oral examination; (e) reported to have systemic inflammatory health or other health conditions, including heart disease, hemophilia or any bleeding disorders; (f) previously been diagnosed with congenital or chronic heart diseases, endocarditis, or rheumatic fever; (g) were undergoing active dialysis treatment; or (h) had undergone cardiovascular disease (CVD) procedures, such as pacemaker or defibrillator implantation, or surgeries involving prosthetic materials on the heart or vessels (e.g., stent placement). Additional exclusion criteria are further described elsewhere (25).

#### 2.1.1 Ascertainment of diabetes status

Participants meeting initial eligibility criteria in pre-clinical phone call screening and providing verbal consent were scheduled for a clinical examination, where they needed to bring documentation, such as physicians’ notes, lab results, or diabetes medication labels to confirm their T2D status. SOALS participants with suspected T2D had glycated hemoglobin (HbA1c) ≥6.5% or fasting blood glucose ≥126 mg/dl (26, 27).

### 2.2 Study procedures

All eligible participants from the pre-clinical screening were scheduled for the clinical examination. Written informed consent, signed by all participants, was obtained prior to conducting any study procedures. The study protocol was approved by the Institutional Review Board of the University of Puerto Rico (IRB # B0930116) and adhered to the principles of the Helsinki Declaration of 1975, as revised in 2013 (25).

### 2.3 Food choices assessment and classification

Dietary habits were measured using a food frequency questionnaire where subjects answered questions about how often they consumed certain foods weekly throughout the past year. There were sixteen different categories of foods. The food frequency options participants were able to select ranged from “less than once per week” to “3 + times per day”, and there were seven options to choose from in total (See Supplementary Figure S1). The questionnaire was designed to assess breakfast, lunch, dinner, and snacks, as well as dining out to ensure the most representative dietary history data of the participants. Food choices were, then, categorized as “healthy” vs. “unhealthy” based on typical health outcomes associated with the corresponding food. Foods categorized as “healthy” included fruits, vegetables, grains, nuts, oats, seeds, and fish. Foods categorized as “unhealthy” included fast food products, chips, desserts (pastries, cakes, cookies, chocolates, etc.), rice and beans cooked with salt and a touch of oil, often sautéed with bacon and seasoned with “sazon”, mofongo, fatty meats, and fried foods.

### 2.4 Periodontal primary outcome

The primary outcome of periodontal status was determined by probing pocket depth (PPD) and bleeding on probing (BOP) at the same tooth. These examinations were done by trained dental professionals at the time of the assessment during the LLIPDS study (28). PPD was measured at six sites per tooth except for the third molars. PPD was measured from the gingival margin to the bottom of the pocket using a periodontal probe. Bleeding on probing was recorded at two of the six sites where PPD measurements were taken, one on the buccal side and one on the lingual side. More detailed information on periodontal assessment is provided elsewhere (25).

### 2.5 Glucose control secondary outcome

The fasting serum glucose and glycosylated hemoglobin (HbA1c%) were measured (29), and HbA1c levels were assessed using a latex immunoagglutination inhibition method with monoclonal antibodies, utilizing a Siemens Kit for DCA 2000 and DCA Vantage Analyzer. An HbA1c measurement was considered uncontrolled if it exceeded ≥7% (27, 28).

### 2.6 Other available data

The LLIPDS questionnaire document from the original study (7) provides a detailed account of all collected data. In addition to information obtained through the food frequency questionnaire, we extracted data on age, gender, educational level (≤12 years vs. >12 years), smoking status (never, former, or current), alcohol consumption (abstainer, former, or current), exercise habits (yes/no), duration of diabetes (years), and comprehensive details of current medication use, all gathered at the time of examination. Anthropometric measurements including waist circumference, height, and weight were recorded to the nearest 0.1 cm or 0.1 kg, and body mass index (BMI) was calculated using weight (in kg) divided by the square of height (in meters). Three blood pressure readings were averaged, and fasting serum insulin and lipid panel measurements were also recorded (29). Oral hygiene status was assessed using the mean plaque index (30) measured at six Ramfjord teeth (31) and self-reported daily tooth brushing frequency (28), while the number of missing teeth was also documented.

### 2.7 Statistical analysis

The independent variable, Healthy Eating Score, was calculated by subtracting the mean unhealthy food consumption from the mean healthy food consumption. A positive result indicated higher consumption of healthy food, while a negative result indicated higher consumption of unhealthy food. The primary outcome was defined as the number of teeth with PPD ≥ 4 mm and BOP at the same tooth, while the secondary outcome was the presence of uncontrolled glucose levels. A summary of the study population's general characteristics was given. The variables were presented as mean (± standard deviation), median (25th, 75th percentiles), or frequency (percent), as appropriate. We examined correlations between covariates, ensuring no highly correlated covariates were included in the same model. We used linear regression models adjusted for age, gender, educational level, smoking status, alcohol consumption, BMI, plaque index, HbA1c, and total cholesterol to estimate the association between food choices and PD, and logistic regression (same model adjustment factors, except PD, replaced hb1c) to estimate glucose control. Crude and adjusted β coefficient or odds ratio (OR), 95% CI, and *p*-value were provided.

## 3 Results

In total, 2,236 participants were called for screening, but only 595 individuals were screened. The remaining 1,641 participants were either ineligible, unreachable, provided an incomplete screening, or moved out of the country (Figure 1). Of the 595 screened, 117 had incomplete visit for various reasons (e.g., not show up for the visit, visit overlapped with work hours, not interested, COVID-19 lockdown), and 211 were ineligible. Out of the 267 eligible participants who completed the visit, 7 were later excluded from the study due to protocol deviations (e.g., multiple visits, T1D), leaving 260 participants with glucose control outcome data. Additionally, 3 participants were excluded from the periodontal outcome analysis due to missing periodontal data, resulting in a sample size of 257. Nineteen participants were further excluded from the statistical analysis due to missing data on key variables, particularly the variable for food frequency consumption.

The general distribution of the Healthy Eating Score, periodontal parameters, and glucose control are described in Table 1. The mean Healthy Eating Score was 0.4 ± 6.6. The mean and median values of number of teeth with PPD ≥ 4 mm and BOP at the same tooth were 2.9 ± 4.6 and 1(0,3) teeth, respectively. The total number of participants (%) with uncontrolled HbA1c determined by levels greater than or equal to 7% was 170 (64.2%) (Table 1).

**Table 1:** Distribution of healthy eating score in the last 12 months prior to the dental visit (*N* = 238) exposure, and number of teeth with PPD ≥ 4 mm and BOP (*N* = 257) and uncontrolled HbA1c (*N* = 260) outcomes.

Exposure or outcome	Mean ± SD Median (q1, q3) Range (min, max), or *N* (%)
Healthy eating score: Mean weekly heathy food consumption—mean weekly unhealthy food consumption	Mean: 0.4 ± 6.6 Median: 0.5 (−3.9, 4.5) Range (−17.5, 22.1)
Number of teeth with PPD ≥ 4 mm and BOP at the same tooth	Mean: 2.9 ± 4.6 Median: 1 (0,3) Range (0,23)
Uncontrolled HbA1c (≥7%)	170 (64.2%)

The general characteristics of Hispanic adults with T2D, stratified by periodontal status, are summarized in Table 2. Participants with at least one tooth affected by PPD ≥ 4 mm and BOP at the same tooth were more likely to be male (51.7% vs. 34.0%, *p* < 0.01), had a lower education level (41.7% vs. 25.5%, *p* < 0.01), exhibited higher mean HbA1c (8.3% ± 2.1 vs. 7.7% ± 1.8, *p* = 0.02), and showed a higher mean plaque index (1.1 ± 0.6 vs. 0.9 ± 0.5, *p* < 0.01), indicating poorer oral hygiene compared to those without any tooth with PPD ≥ 4 mm and BOP at the same tooth. Interestingly, those with poorer periodontal outcomes reported slightly worse food choices (median Healthy Eating Score of −0.7 vs. 2.1, *p* = 0.03) compared to those without affected teeth.

**Table 2:** General characteristics of Hispanic adults with type 2 diabetes by periodontal status, *N* = 257.

Characteristic	No tooth with PPD ≥ 4 mm and BOP (i.e.,<1 median) *n* = 106 (41.25%)	≥1 tooth with PPD ≥ 4 mm and BOP (i.e., ≥ 1 median) *n* = 151 (58.75%)	*p*-value
	Mean ± SD, Median (q1, q3), *N* (%)	Mean ± SD, Median (q1, q3), *N* (%)	
Age (years)	54.3 ± 5.8	54.5 ± 6.1	0.784
Male gender	36 (34.0)	78 (51.7)	**0**.**005**
Education (≤12 years)	27 (25.5)	63 (41.7)	**0**.**007**
Smoking status			0.349
Never	72 (68.6)	92 (60.9)	
Former	21 (20.0)	42 (27.8)	
Current	12 (11.4)	17 (11.3)	
Alcohol drinking (Current)^a^	42 (39.6)	67 (45.3)	0.370
Hypertension (yes)	72 (67.9)	99 (65.6)	0.693
BMI (kg/m^2^)	34.5 ± 9.4	35.4 ± 11.5	0.571
HbA1c (%)	7.7 ± 1.8	8.3 ± 2.1	**0**.**015**
Total-Cholesterol (mg/dl)	173.8 ± 43.0	177.5 ± 44.7	0.510
HDL-Cholesterol (mg/dl)^b^	48.4 ± 13.3	46.1 ± 13.4	0.189
Diabetes duration (years)^a^	9.9 ± 6.9	11.5 ± 8.4	0.121
Taking Metformin medication	43 (40.6)	84 (55.6)	**0**.**017**
LLA current users (yes)^c^	58 (54.7)	78 (51.7)	0.628
LLA duration of use (years)^c^	0.3 (0, 3)	0.1 (0,5)	0.169
Anti-inflammatory agents (yes)^a^	9 (8.5)	8 (5.3)	0.311
Mean Plaque index^a^	0.9 ± 0.5	1.1 ± 0.6	**0**.**004**
Tooth brushing (≥twice/day)	19 (17.9)	26 (17.5)	0.922
Dental flossing (≥1 a day)	38 (35.9)	61 (40.9)	0.411
Dental visits (≥1 in the past year)	28 (26.4)	54 (36.2)	0.098
Missing teeth	2.6 ± 3.4	2.2 ± 2.4	0.815
Diet habits	2.1 (−3.5, 6.3)	−0.7 (−4.5, 4.0)	**0**.**030**
Exercise (yes)^a^	32 (30.5)	46 (30.7)	0.974

^a^ Missing values, diabetes duration 11, alcohol consumption 2; plaque index 7, exercise 2; ^b^HDL-cholesterol, high-density lipoprotein-cholesterol; ^c^LLA, lipid-lowering agents.

*P-*values in bolds are statistically significant (*p* < 0.05).

Notably, though not statistically significant, a higher frequency of dental flossing (40.9% vs. 35.9%) and more frequent dental visits in the past year (36.2% vs. 26.4%) suggested better oral health behaviors among participants with poorer periodontal outcomes compared to those without affected teeth. These behaviors might have reflected an adaptive response to the severity of their periodontal condition, as observed in previous research using the same dataset (14).

Variables such as age, smoking status, alcohol consumption, BMI, total cholesterol, HDL levels, diabetes duration, use of anti-inflammatory agents, and oral health behaviors, such as frequency of tooth brushing, did not show significant differences between groups.

Table 3 displays the general characteristics of Hispanic adults with T2D based on glucose control. Participants with uncontrolled glucose (HbA1c levels ≥7%) were younger (53.8 ± 6.0 vs. 55.6 ± 5.7 years, *p* = 0.02), more likely to be current or former smokers (current: 13.6% vs. 6.7%; former: 28.4% vs. 16.7%, *p* = 0.01), had higher total cholesterol (179.4 ± 47.4 vs. 168.4 ± 35.9 mg/dl, *p* = 0.06), and lower high-density lipoprotein cholesterol levels (45.8 ± 13.2 vs. 49.5 ± 13.4 mg/dl, *p* = 0.03). They also had a longer duration of diabetes (11.6 ± 7.6 vs. 9.4 ± 8.1 years, *p* = 0.041) compared to participants with controlled glucose (HbA1c levels <7%).

**Table 3:** General characteristics of Hispanic adults with type 2 diabetes by glucose control, *N* = 260.

Characteristic	HbA1c < 7% *n* = 90 (34.6%)	HbA1c ≥ 7% *n* = 170 (65.4%)	*p*-value
	Mean ± SD, Median (q1, q3), *N* (%)	Mean ± SD, Median (q1, q3), *N* (%)	
Age (years)	55.6 ± 5.7	53.8 ± 6.0	**0**.**023**
Male gender	36 (40.0)	81 (47.7)	0.238
Education (≤12 years)	30 (33.3)	60 (35.3)	0.752
Smoking status			**0**.**011**
Never	69 (76.7)	98 (58.0)	
Former	15 (16.7)	48 (28.4)	
Current	6 (6.7)	23 (13.6)	
Alcohol drinking (Current)^a^	34 (38.2)	76 (45.2)	0.278
Hypertension (yes)	61 (67.8)	111 (65.3)	0.687
BMI (kg/m^2^)	36.2 ± 12.3	34.3 ± 9.6	0.179
Taking Metformin medication	48 (53.3)	79 (46.5)	0.292
Total-cholesterol (mg/dl)	168.4 ± 35.9	179.4 ± 47.4	0.056
HDL-cholesterol (mg/dl)^b^	49.5 ± 13.4	45.8 ± 13.2	**0**.**033**
Diabetes duration (years)^a^	9.4 ± 8.1	11.6 ± 7.6	**0**.**041**
LLA current users (yes)^c^	44 (48.9)	93 (54.7)	0.371
LLA duration of use (years)^c^	0 (0, 3)	0.2 (0, 4.7)	0.601
Anti-inflammatory agents (yes)^a^	8 (8.9)	9 (5.3)	0.265
Mean Plaque index^a^	1.0 ± 0.5	1.1 ± 0.6	0.350
Tooth brushing (≥twice/day)	21 (23.3)	25 (14.9)	0.091
Dental flossing (≥1 a day)	29 (32.2)	72 (42.9)	0.095
Dental visits (≥1 in the past year)	27 (30.0)	56 (33.3)	0.585
Teeth with PPD ≥ 4 mm and BOP (*n*)	0 (0,3)	1 (0,4)	0.060
Missing teeth	2.6 ± 3.1	2.3 ± 2.7	**0**.**011**
Bleeding on probing (%)	22.7 ± 11.9	22.1 ± 12.2	0.910
Healthy eating score	1.9 (−3.5, 4.6)	−0.5 (−4.2, 4.4)	**0**.**042**
Exercise (yes)^a^	32 (36.0)	47 (27.8)	0.177

^a^ Missing values, diabetes duration 11, alcohol consumption 2; plaque index 7, exercise 2; ^b^HDL-cholesterol, High-density lipoprotein-cholesterol; ^c^LLA, lipid-lowering agents.

*P-*values in bold are statistically significant (*p* < 0.05).

Notably, the mean Healthy Eating Score was significantly lower in the uncontrolled glucose group (median: −0.5 vs. 1.9, *p* = 0.04). Additionally, participants with worse glucose control had fewer missing teeth (2.3 ± 2.7 vs. 2.6 ± 3.1 teeth, *p* = 0.01) but tended to have a greater number of teeth with PPD ≥ 4 mm and BOP (median: 1 vs. 0 tooth, *p* = 0.06), though this latter finding was borderline significant compared to the controlled glucose group.

Interestingly, though not statistically significant, participants with worse glucose control reported more frequent dental visits (33.3% vs. 30.0%) and flossing (42.9% vs. 32.2%) compared to those with controlled glucose, possibly as a response to discomfort from their periodontal condition.

Insignificant results were found in this analysis with the following variables: male gender, education, alcohol status, hypertension, BMI, LLA use and duration, anti-inflammatory agent use, mean plaque index, frequency of tooth brushing, bleeding on probing percentage, and exercise.

Table 4 (See also Supplementary Figure S2) displays the Regression coefficient (β, SE) for the association between Healthy Eating Score and the natural logarithm of the number of teeth with PPD ≥ 4 mm and BOP at the same tooth + 1. The Healthy Eating Score showed an adjusted β of −0.02 (SE = 0.01, 95% confidence interval (CI) of −0.04 to −0.001 (*p* = 0.035), indicating a small but significant association between healthier eating and better periodontal outcomes. For gender, the adjusted regression coefficient was 0.28 (SE = 0.13, 95% CI: −0.53 to −0.03, *p* = 0.026), suggesting that being male is significantly associated with more negative periodontal outcomes. Education level showed an adjusted β of −0.42 (SE = 0.13, 95% CI: −0.67 to −0.17, *p* *=* 0.001), indicating that lower education is associated with worse periodontal health. Higher plaque index values were significantly associated with worse periodontal outcomes, with an adjusted β of 0.32 (SE = 0.11, 95% CI: 0.09–0.54, *p* = 0.006). Age, smoking status, alcohol status, BMI, and HbA1c did not show significant associations in this analysis.

**Table 4:** Regression coefficient (β, SE) for the association of healthy eating score with number of teeth with PPD ≥ 4 mm and BOP at the same tooth.

Independent variable and covariates	Crude			Adjusted^a^		
	β (SE)	95% CI	*P*-value	β (SE)	95% CI	*P*-value
Healthy Eating Score	−0.03 (0.01)	(−0.05; −0.01)	0.009	−0.02 (0.01)	(−0.04; −0.001)	**0.035**
Age				−0.01 (0.01)	(−0.03; 0.01)	0.504
Gender				−0.28 (0.13)	(−0.53; −0.03)	**0.026**
Educational level				−0.42 (0.13)	(−0.67; −0.17)	**0.001**
Smoking Status				−0.13 (0.22)	(−0.57; 0.31)	0.566
Alcohol Status				−0.06 (0.12)	(−0.29; 0.18)	0.638
BMI				0.001 (0.004)	(−0.01; 0.01)	0.749
Mean plaque Index				0.32 (0.11)	(0.09; 0.54)	**0.006**
HbA1c				−0.01 (0.03)	(−0.07; 0.05)	0.736

*P*-values in bold are statistically significant (*p* < 0.05).

^a^ Linear regression models (with robust SE) adjusted for age, gender, educational level, smoking status, alcohol consumption, BMI, plaque index, and HbA1c.

**Figure 2:**
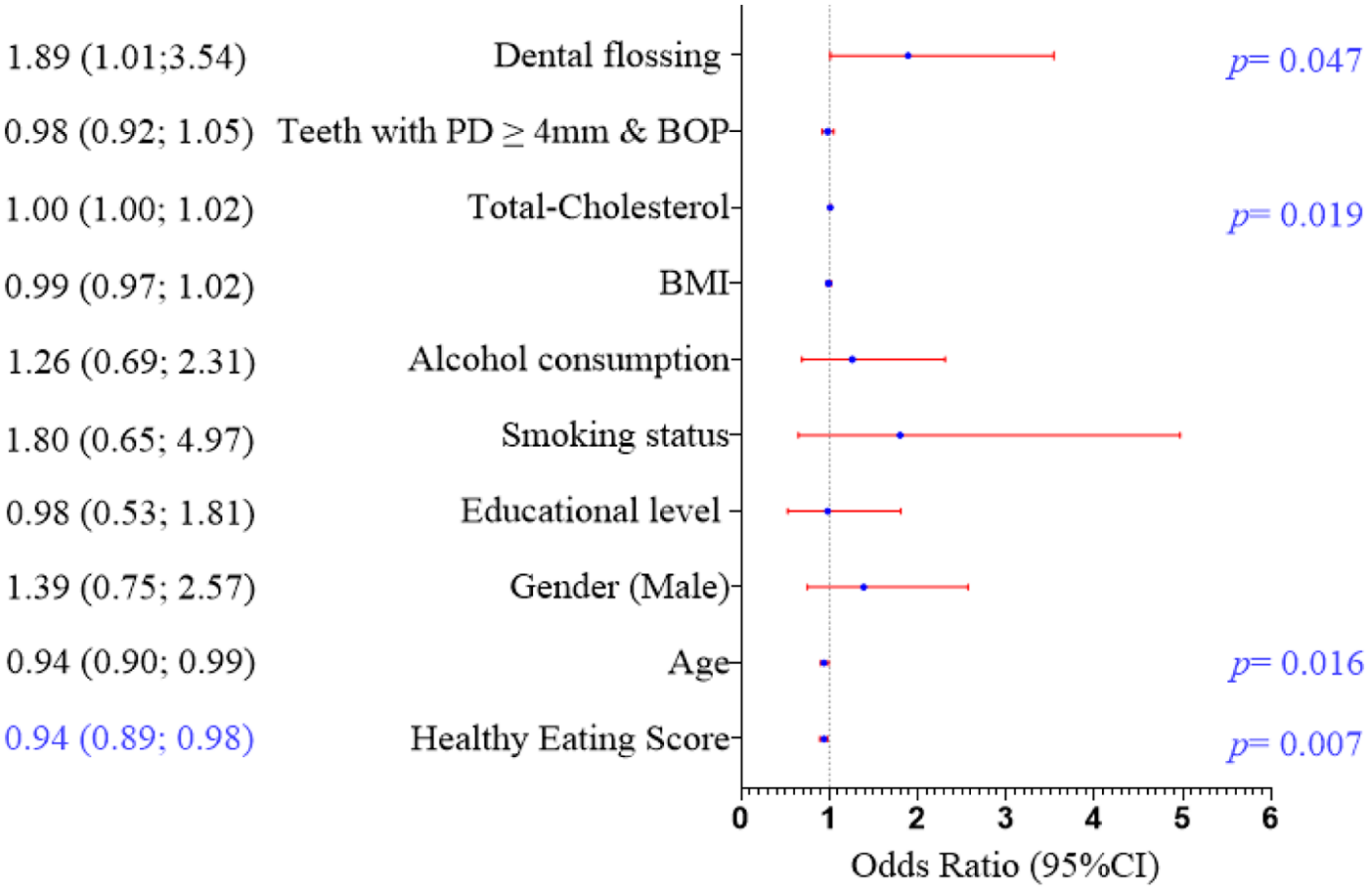
Odds ratio (95%CI) for the association of healthy eating score with uncontrolled glucose (HbA1c).

Figure 2 (see also Supplementary Table S1) displays the Odds Ratios (ORs) with 95% confidence intervals (CIs) for the association between Healthy Eating Score and uncontrolled glucose (HbA1c). The Healthy Eating Score showed an adjusted OR of 0.94 (95% CI: 0.89–0.98, *p* = 0.007), indicating that each one-unit increase in the Healthy Eating Score (i.e., healthier food choices) is associated with a 6% higher likelihood of better glucose control in Hispanic adults with T2D. Age showed an adjusted OR of 0.94 (95% CI: 0.90–0.99, *p* = 0.016), suggesting a slight decrease in the odds of uncontrolled glucose with increasing age. Total cholesterol levels had an adjusted OR of 1.01 (95% CI: 1.00–1.02, *p* = 0.019), indicating a small but significant association with worse glucose control as cholesterol levels increase. Frequency of dental floss had an adjusted OR of 1.89 (1.01–3.54, *p* = 0.047). The following variables were not significantly associated with glucose control in this analysis: gender, educational level, smoking status, alcohol consumption, BMI, and the number of teeth with PPD ≥ 4 mm and BOP.

## 4 Discussion

We found that self-reported food choices categorized as healthier food consumption, compared to unhealthy food consumption, was associated with reduced periodontal inflammation and better glucose control in Hispanic adults with T2D. A healthy diet is known to play a role in preventing or alleviating symptoms of many human diseases. Few studies have assessed diet as a whole regarding its impact on PD progression. Zare et al. conducted a study that demonstrated that Omega-3 Fatty Acid enriched cranberry juice decreased periodontal inflammation, appearance, and glycated hemoglobin levels in patients with T2D. Polyphenolic compounds are found in cranberry extracts, which have shown positive impacts on human health (32). In their study, all cases of periodontitis were scored as less severe following the cranberry juice and/or cranberry juice enriched with Omega-3 FAs (32). This is one of the very few studies available that analyze dietary effects on PD, however it only examined the effects of cranberry juice and Omega-3 FAs instead of overall diet in patients with T2D.

Although the study was not specifically conducted on a diabetic population, Altun et al., studied dietary patterns from 6,209 participants in the Hamburg City Health Study (HCHS). The authors revealed that greater adherence to the ‘Dietary Approaches to Stop Hypertension (DASH) or Mediterranean diet is associated with a lower likelihood of PD (33), as classified by the CDC/AAP definition (34). The DASH diet is a well-established therapeutic diet consisting of fruits, vegetables, and low-fat dairy products.

The National Cancer Institute's diet survey, completed by 923 participants using a validated diet history questionnaire, found no link between overall diet quality [measured by Alternative Healthy Eating Index (AHEI) scores and A Prior Diet Quality Scores (APDQS)] and periodontal parameters, such as mean PPD, mean CAL, and periodontitis (35). However, better adherence to APDQS was associated with reduced percent of sites with BOP (%BOP) (36). Specifically, higher nut consumption was associated with lower mean probing depth (MPD) (*p* = 0.03) and reduced periodontitis, whereas increased red meat intake correlated with higher MPD (*p* = 0.01). Both red meat and trans-fatty acid consumption were associated with higher % BOP (*p* = 0.05).

A systematic review and meta-analysis of previous clinical trials assessing the effects of specific foods or nutrients indicated moderate positive effects of green tea on gingivitis and periodontitis (37). Additionally, a randomized controlled, four-week pilot study suggested that a diet low in carbohydrates but rich in omega-3 fatty acids, vitamin C and D, antioxidants, and fibers could reduce both gingivitis and periodontitis (38).

On the other hand, previous research has shown that unhealthy dietary patterns worsen T2D markers and increases uncontrolled glucose levels (39–44). Our data on Hispanic adults with T2D supports this, demonstrating that both PD and T2D share similar risk factors, including dietary factors, confirming their comorbidity. Moreover, uncontrolled glucose was significantly higher in those who had more severe periodontal assessments (Table 2), or vice versa though the latter association is borderline statistically significant (Table 3), suggesting a connection between the two outcomes. In a small-scale randomized clinical trial involving 30 individuals with T2D, participants were assigned to a strict T2D diet in either a test group following an Okinawan-based Nordic diet (OBND) or a control group receiving standard hospital care over one month. The results showed a reduction in serum HbA1c and BOP scores with no significant differences between the groups (45).

Dietary factors are pivotal in the pathogenesis of both PD and T2D, largely due to their impact on inflammation. Mechanistically, oxidative stress, a key regulator of inflammation, is influenced by dietary patterns and infections (46). Diets abundant in complex carbohydrates are typically beneficial, while those high in refined carbohydrates can contribute to chronic inflammation (46–48). Specifically, high-calorie diets featuring refined and processed foods with elevated postprandial levels of glucose and lipids lead to an imbalance where reactive oxygen species (ROS) production surpasses endogenous antioxidant capabilities, resulting in oxidative stress (46, 47, 49, 50).

The small-scale randomized clinical trial comparing the Okinawan-based Nordic diet (OBND) with standard hospital care over one month, as described above, revealed that serum levels of inflammatory markers—including IFN-γ (interferon gamma), Eotaxin, IL-9 (interleukin-9), IL-17A, IP-10 (interferon-inducible protein-10), MCP-1 (monocyte chemoattractant protein-1), and PDGF-BB (platelet-derived growth factor subunit B)—decreased only in the OBND group (45). In addition, data from the NHANES showed a link between dietary inflammatory index (DII) scores, used to measure diet-related inflammation, and periodontal parameters, such as mean PPD and mean CAL. Furthermore, the association between these metrics and systemic inflammatory markers—white blood cells and segmented neutrophils—was mediated by DII (51), suggesting that an inflammatory diet may play a role in PD development.

In fact, our recent findings from the same current database suggested cholesterol-lowering agents use (LLA) within 1–4 years or taking high intensity statins to be associated with lower level of periodontal parameters among participants with T2D (25, 52). In addition, LLA use within 1–4 years or high-intensity statins correspondently related to lower serum level of soluble vascular cell adhesion molecule-1 (sVCAM-1) and lower gingival crevicular fluid (GCF) levels of IL-1α, soluble intercellular adhesion molecule-1 (sICAM-1), or sVCAM-1 among those participants (52, 53). Also, our previous findings from a longitudinal study has demonstrated moderate or high level of high-density lipoprotein-Cholesterol (HDL-C) to significantly reduce the risk of PD development among overweight or obese Hispanic individuals (54).

In addition to well-established diets such as the Mediterranean, DASH, vegetarian, and Okinawa diets, which are known to reduce the risk of chronic diseases like T2D (55–60), the emerging planetary health diet, which focuses on promoting sustainable and nutritious food choices, may also benefit both PD and T2D (61). This diet emphasizes environmentally friendly foods that support human health, with evidence linking plant-based diets and reduced processed food consumption to improved health outcomes (62, 63). Integrating this approach into dietary recommendations could not only improve both oral and systemic health but also contribute to environmental sustainability (64).

Beyond our findings, broader perspectives on the connection between dietary habits, PD, and T2D should be considered. Recent studies suggest that nutrition is a shared risk factor for both periodontal and chronic diseases, with poor nutrition potentially influencing periodontal health early in life and contributing to the later development of chronic conditions like T2D (60). This highlights that changes in periodontal health, particularly in early life, may serve as an early warning sign, signaling the need for improved dietary quality to reduce the risk of developing T2D or other CVD later in life.

Our study highlights the role of health behaviors, such as diet, in the prevalence of PD among individuals with T2D. Male gender, lower education level, and a higher mean plaque index were also independently associated with higher PD. Previous studies suggest that men and those with lower education levels tend to engage in poorer health behaviors and are more prone to chronic diseases (28, 65), which aligns with our findings, and has been observed in PD studies (14, 25, 28, 66, 67). Dental plaque may reflect oral hygiene status, with our previous studies linking a higher plaque index to worse periodontal parameters (25, 66). Moreover, supragingival plaque has been reported to serve as a reservoir for putative periodontal pathogens, potentially facilitating their spread to subgingival sites (67). These pathogens are recognized as key risk factors for PD (68).

Notably, participants with poorer glucose control (higher HbA1c) exhibited paradoxical health behaviors—such as more frequent dental visits and flossing—likely due to the discomfort from PD. This might reflect an adaptation to the severity of their condition. Our multivariable analysis revealed that while healthier dietary habits and older age were associated with better glucose control, higher cholesterol and increased dental flossing frequency were linked to worse control. These findings suggest a potential bidirectional relationship between oral health and diabetes management.

Despite its cross-sectional design, our model highlights the significant role of diet in PD and glucose control, independent of factors such as age, gender, education, and others. It also underscores the influence of socioeconomic status (SES) and health awareness on oral and metabolic health, suggesting that diet, SES, and other behavioral factors collectively impact outcomes. Future studies should explore additional factors, including detailed assessments of physical activity and other health behaviors, to better understand these complex interactions.

To the best of our knowledge, this study is the first to examine the potential association between dietary habits and PD among Hispanics with T2D. It is also the second study, following the small-scale OBND clinical trial mentioned earlier, to investigate the diet as a risk factor for both PD and glucose control in T2D, highlighting their common dietary risk factor in comorbid conditions.

We utilized a concise 16-item FFQ to gather dietary habit data from participants over the year preceding their visit. Since this was a post-study classification, participants could not falsely report food consumption information at the time of the assessment. The FFQ focused on estimating the frequency of consumption rather than quantifying precise food amounts, which could introduce significant measurement errors given the variability over a year. However, it effectively captured dietary patterns and potential correlations between food groups, offering insights into dietary patterns that may relate to chronic metabolic conditions (69–73).

Our study generated reliable data on blood glucose and lipid panel measurements, as well as comprehensive periodontal assessments conducted according to the NHANES oral health protocol (74). Additionally, we gathered extensive information on factors potentially linked to periodontal health and glucose regulation, which can be used to generate further hypotheses. Our findings may offer valuable insights into public health challenges specific to Hispanic individuals with T2D.

The limitations of the database used in this study are detailed in previous publications (25, 66). Briefly, this study utilized a non-probability convenience sample, which means the findings cannot be generalized beyond the diabetic population residing in Puerto Rico. However, an analysis of the main characteristics of the study population by the source of participants (i.e., 50% from PRCD, 45% from the general population, and 5% from COSSMA), including age, gender, education, and smoking status, revealed no significant differences.

Recall bias may have occurred in the collection of dietary habits and other potential confounding factors, such as medication use, dosages, and duration, from interview-based questionnaires. To mitigate this, we utilized thorough but straightforward questions designed to enhance participants’ recall without introducing bias by clarifying the purpose of the questions.

The cross-sectional study design limits our ability to make causal interpretations due to the temporal sequence issue. Participants might have had elevated levels of the periodontal primary outcome long before the dietary data were collected, although this is less likely with the glucose control secondary outcome. Given the limited scope of our data, we cannot definitively determine the actual temporal sequence.

Our findings have significant public health implications at the intersection of studies in promoting oral and metabolic health. Choosing nutrient-dense foods that are also typically high in antioxidants can assist in managing and preventing uncontrolled glucose levels and simultaneously aid in the prevention or management of PD, as both conditions are interrelated through diet and inflammation. The oral cavity often reflects overall health, and the presence of PD may indicate uncontrolled glucose levels in individuals with T2D.

## 5 Conclusion

In summary, our cross-sectional study underscores the association between a healthier dietary pattern, reduced periodontal inflammation, and improved glucose control among Hispanics with T2D. While diet plays a key role in both periodontal health and glucose control, other behavioral and demographic factors, such as SES, gender, and health awareness, likely have an even more significant impact. Our findings highlight that future studies should explore these relationships further, considering additional factors like physical activity and other health behaviors, which may further inform the development of targeted interventions.

Given the potential interconnection between nutrition, chronic disease prevention, and planetary health, future research should also investigate the role of sustainable dietary patterns in managing T2D and PD, particularly in underserved populations. By addressing both individual and environmental health concerns, such studies may offer valuable insights into comprehensive, long-term health solutions.

To substantiate these findings, future research will require extensive prospective studies with larger sample sizes and validated dietary assessment tools tailored to the study population. While our study's cross-sectional design limits causal inference, it suggests that a healthy diet may help prevent or manage PD in addition to improving T2D outcomes, while routine oral hygiene remains essential for controlling dental plaque biofilm. Moreover, our results support the connection between T2D and PD, reinforcing the influence of food choices on both conditions. Future intervention studies aimed at modifying dietary patterns and behavioral factors to enhance glycemic control may also foster improved oral health and overall well-being within the Hispanic population.

## Data Availability

The datasets presented in this article are not readily available because the data and/or materials presented in this study are available on request from the co-author. The data is not publicly available due to privacy and ethical restrictions. Requests to access the datasets should be directed to Oelisoa M. Andriankaja, oelisoa.andriankaja@uky.edu.
