# Supplementary material for: Cross-sectional association among dietary habits, periodontitis, and uncontrolled diabetes in Hispanics: the LLIPDS study
Source: Front Oral Health. 2025 Jan 31;6:1468995. doi: 10.3389/froh.2025.1468995 (PMC11825391; doi:10.3389/froh.2025.1468995)
Supplement: Supplementary file 1 [file Datasheet1.docx]

Supplementary Material

**Figure S1.** Dietary habits excerpt from questionnaire given to participants from LLIPDS study (2017-2020)


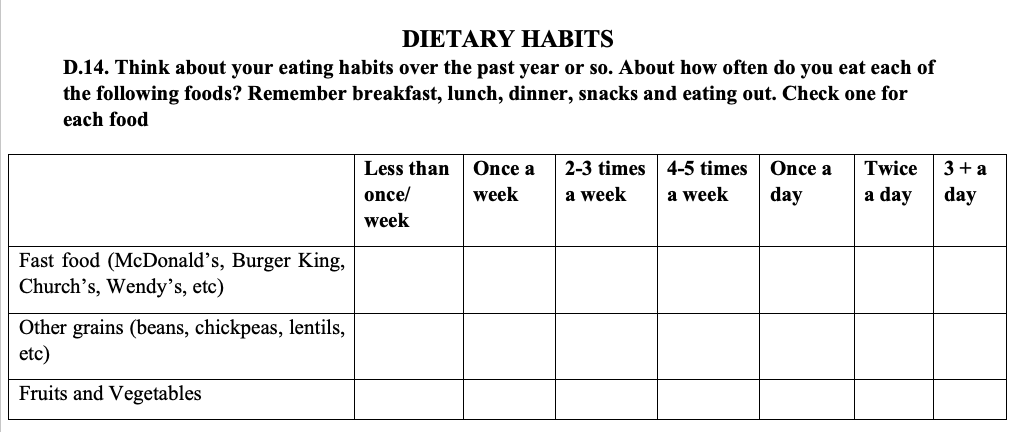


**Figure S2.** Regression coefficient (β, SE) for the association of Healthy Eating Score with a natural logarithm of (number of teeth with PPD ≥ 4mm and BOP at the same tooth + 1)


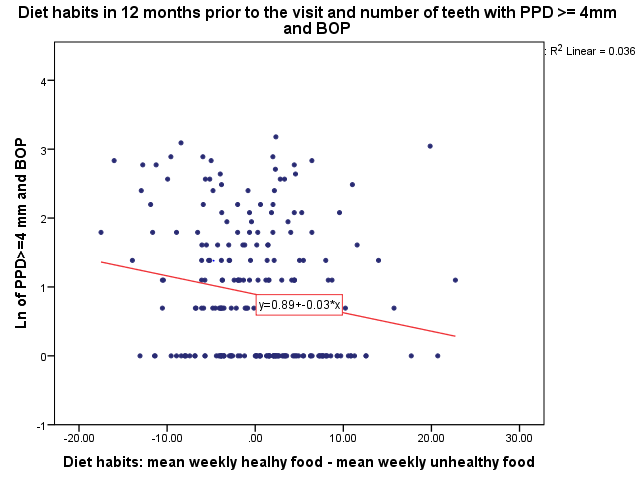


**Table S1.** Odds Ratio (95%CI) for the association of Healthy Eating Score with uncontrolled

glucose (HbA1c)

|  | Crude | | | Adjusted* | | |
| --- | --- | --- | --- | --- | --- | --- |
| Independent variable  and covariates | OR | 95% CI | *P*-value | OR | 95% CI | *P*-value |
| Healthy Eating Score | 0.96 | (0.92; 1.00) | 0.031 | 0.94 | (0.89; 0.98) | **0.007** |
| Age |  |  |  | 0.94 | (0.90; 0.99) | **0.016** |
| Gender (male) |  |  |  | 1.39 | (0.75; 2.57) | 0.294 |
| Educational level |  |  |  | 0.98 | (0.53; 1.81) | 0.937 |
| Smoking Status |  |  |  | 1.80 | (0.65; 4.97) | 0.257 |
| Alcohol Status |  |  |  | 1.26 | (0.69; 2.31) | 0.446 |
| BMI |  |  |  | 0.99 | (0.97; 1.02) | 0.579 |
| Total-Cholesterol |  |  |  | 1.01 | (1.00;1.02) | **0.019** |
| Dental flossing (≥ 1 a day) |  |  |  | 1.89 | (1.01; 3.54) | **0.047** |
| Number of teeth with PPD≥ 4mm and BOP |  |  |  | 0.98 | (0.92; 1.05) | 0.551 |

* Logistic regression models (OR, 95%CI) adjusted for age, gender, educational level, smoking status, alcohol consumption, BMI, Total-Cholesterol, and number of teeth with PPD ≥ 4mm and BOP.
